# DHCT-GAN: Improving EEG Signal Quality with a Dual-Branch Hybrid CNN–Transformer Network

**DOI:** 10.3390/s25010231

**Published:** 2025-01-03

**Authors:** Yinan Cai, Zhao Meng, Dian Huang

**Affiliations:** 1 National Supercomputing Center in Shenzhen, Shenzhen 518055, China; caiyinans@163.com; 2 University of Chinese Academy of Sciences, Beijing 100049, China; mzhaowl@163.com; 3 Guangdong Institute of Intelligence Science and Technology, Hengqin, Zhuhai 519031, China

**Keywords:** electroencephalogram (EEG), transformer, generative adversarial network (GAN), denoising

## Abstract

Electroencephalogram (EEG) signals are important bioelectrical signals widely used in brain activity studies, cognitive mechanism research, and the diagnosis and treatment of neurological disorders. However, EEG signals are often influenced by various physiological artifacts, which can significantly affect data analysis and diagnosis. Recently, deep learning-based EEG denoising methods have exhibited unique advantages over traditional methods. Most existing methods mainly focus on identifying the characteristics of clean EEG signals to facilitate artifact removal; however, the potential to integrate cross-disciplinary knowledge, such as insights from artifact research, remains an area that requires further exploration. In this study, we developed DHCT-GAN, a new EEG denoising model, using a dual-branch hybrid network architecture. This model independently learns features from both clean EEG signals and artifact signals, then fuses this information through an adaptive gating network to generate denoised EEG signals that accurately preserve EEG signal features while effectively removing artifacts. We evaluated DHCT-GAN’s performance through waveform analysis, power spectral density (PSD) analysis, and six performance metrics. The results demonstrate that DHCT-GAN significantly outperforms recent state-of-the-art networks in removing various artifacts. Furthermore, ablation experiments revealed that the hybrid model surpasses single-branch models in artifact removal, underscoring the crucial role of artifact knowledge constraints in improving denoising effectiveness.

## 1. Introduction

Electroencephalography (EEG) is a non-invasive neurophysiological technique that measures and records the electrical activity of the brain [1]. It is widely used in clinical diagnosis [2] and research on neuroscience, cognitive neuroscience [3,4,5,6], and brain–computer interface (BCI) [7,8,9]. However, EEG signals are easily contaminated by various artifacts [10,11,12,13]. Artifacts in EEG signals can introduce significant biases, which may lead to the misinterpretation of essential EEG characteristics [14,15], potentially impact clinical diagnoses of Alzheimer’s disease [16], and affect the performance of BCI systems [17]. Artifacts stemming from non-physiological (e.g., faulty electrodes, line noise, and high electrode impedance) and non-brain physiological (e.g., electrocardiography (ECG), electrooculography (EOG), and electromyography (EMG)) sources can be minimized through the use of precise recording systems and strict recording protocols [18]. However, in practical applications, such ideal conditions are often not achievable. Additionally, physiological artifacts like ECG, EMG, and EOG closely resemble pure EEG signals in characteristics such as amplitude and frequency range [19,20,21], making their removal particularly challenging. Therefore, there is a critical need to develop effective artifact removal techniques to suppress these interferences and preserve the authenticity of EEG signals.

Several traditional approaches for EEG denoising have been developed and demonstrate promising results. These include regression [22], filtering [23,24], the wavelet transform algorithm (WVT), empirical-mode decomposition (EMD) [25,26], and blind source separation (BSS) [27,28,29,30,31,32,33,34,35]. To leverage the strengths of each method, strategies such as EMD-BSS [36,37,38] and WVT-BSS [39,40,41] have been proposed to improve accuracy. However, these approaches have certain drawbacks in practical applications. For example, the absence of external reference channels in regression algorithms [22] limits their effectiveness in removing EOG and ECG artifacts [42]. Filtering may inadvertently discard valuable information [43,44]. EMD algorithms, though effective, are complex and time-consuming. BSS algorithms are vulnerable to rank-deficient data, a common issue arising from practices such as re-referencing and channel interpolation, which limits their effectiveness [45]. In general, most traditional methods have difficulty in effectively eliminating noise from EEG signals, particularly in the absence of reference channels or when manual inspection is required.

Recently, researchers are increasingly turning to deep learning methods to remove artifacts from EEG signals. Neural network architectures based on denoising autoencoders (DAEs), long short-term memory networks (LSTM), and convolutional neural networks (CNNs) are widely used [46,47,48]. For instance, Leite et al. [49] demonstrated the efficacy of DAEs in removing artifacts such as eye blinks and jaw clenching. Xiong et al. [50] proposed a dual-pathway autoencoder (DPAE) framework to capture EEG features at multiple scales for improved denoising. Sun et al. [51] developed a one-dimensional residual convolutional neural network (1D-ResCNN) that effectively removes artifacts while preserving signal clarity. Xiong et al. [52] introduced an algorithm named DWINet, which utilizes the image-dehazing capability of DRHNet to enhance the denoising performance of EEG signals. Gao et al. [53] introduced a method called DuoCL, which combines CNN and LSTM models for deep artifact removal. Wu et al. [54] proposed a neural architecture search with large kernels to enhance denoising performance. Huang et al. [55] developed LTDNet-EEG, a lightweight network designed for real-time EEG signal denoising, and optimized both model structure and computational efficiency. Pei et al. [56] proposed DTP-Net, which reconstructs EEG signals in the time–frequency domain by reusing multi-scale features. Wang et al. [57] deployed the Retentive Network architecture (EEGDiR) for EEG denoising, and exploited its robust feature extraction and comprehensive modeling prowess.

Additionally, Generative Adversarial Networks (GANs), as representatives of adversarial training, have demonstrated excellent performance in EEG signal denoising [58,59]. For instance, Brophy et al. [60] employed GAN to remove EMG and EOG artifacts, while Gandhi et al. [61] designed an asymmetric GAN to denoise EEG time series data. Sumiya et al. [62] utilized GANs to denoise mouse EEG signals. Sawangjai et al. [17] proposed EEGANet, a GAN-based framework for removing ocular artifacts in multi-channel EEG data. Dong et al. [63] developed a novel EEG denoising method based on Wasserstein GAN (WGAN), which effectively decomposes, detects, and removes artifacts from EEG signals.

Recently, Transformer architecture has shown powerful capabilities in EEG denoising [64,65,66]. For example, Pu et al. [67] proposed EEGDNet, a Transformer-based network that removes EOG and EMG artifacts. Furthermore, Yin et al. [68] developed GCTNet, a novel network that combines GAN, parallel CNN, and Transformer architectures, which outperforms many advanced networks in various artifact removal tasks. Huang et al. [69] introduced EEGDfus, a conditional diffusion model for fine-grained EEG denoising, which demonstrates excellent performance in practical applications.

Although deep learning models have shown significant improvements in EEG denoising, there are still some limitations. First, EEG signals are often contaminated by diverse artifacts with varying characteristics, and most previous denoising approaches focus solely on noise removal without learning artifact features, leading to unstable performance and poor handling of varied artifact types. In addition, previous deep learning methods have been limited in their ability to model spatiotemporal features effectively. For instance, CNNs capture local spatial features well but fail to account for long-term temporal dependencies, while Transformer architectures excel at modeling long-range dependencies but underperform in leveraging local features, limiting their effectiveness in fine-grained artifact removal. Moreover, GAN-based structures, while promising, suffer from issues like mode collapse and unstable output during training, particularly with complex artifacts. The reliance on a single discriminator further exacerbates instability, affecting overall denoising performance.

Motivated by the aforementioned challenges, this research introduces DHCT-GAN, a dual-branch hybrid CNN–Transformer network for EEG denoising. The main innovations of DHCT-GAN are as follows:

1. Dual-branch architecture for artifact-specific learning: This network features two branches, one for learning clean EEG features and the other for artifact features. This design allows the model to distinguish EEG signals and artifacts, leveraging cross-domain knowledge for more effective denoising. A gated network module controls the information exchange between branches, adapting to diverse data characteristics.
2. Multi-scale feature extraction and dependency capture: By combining CNNs and Transformers, DHCT-GAN captures both spatial and temporal characteristics, enhancing adaptability to complex signal dynamics. The integration of global and local Transformers enables the model to capture long-term and short-term dependencies, boosting feature robustness.
3. Multi-discriminator GAN framework for stability: DHCT-GAN employs a multi-discriminator approach to mitigate issues such as mode collapse, ensuring stable and reliable denoising across diverse artifacts.

In this study, the denoising capabilities of DHCT-GAN were evaluated for four types of artifacts—EMG, EOG, ECG, and mixed (EMG + EOG)—using three publicly available datasets. A comparative analysis was conducted with several recent state-of-the-art EEG denoising methods. Quantitative evaluation using six performance metrics demonstrated that DHCT-GAN outperforms the current leading methods, showcasing its superior effectiveness and robustness.

The main contributions of this paper are summarized as follows:

1. We introduce a dual-branch hybrid CNN–Transformer network specifically designed for EEG denoising. This network explicitly models the noise and incorporates it as conditional information, effectively extracting both local and global features from EEG signals, enabling a more accurate and flexible denoising process. Additionally, the use of a multi-discriminator approach enhances the stability of model training.
2. The design of DHCT-GAN carefully addresses the diversity and variability of artifact challenges, making it highly effective in practical scenarios, especially in areas such as BCI and clinical EEG analysis.
3. The effectiveness and robustness of DHCT-GAN were validated on three public datasets. Experimental results demonstrate its ability to effectively remove four types of artifacts and reconstruct more accurate waveforms. Quantitative evaluation further highlights its superior performance compared to recent state-of-the-art models.

The structure of this article is as follows: Section 2 provides a comprehensive introduction to the proposed network, including the dataset and evaluation metrics used in this study. Section 3 demonstrates the denoising results of the proposed network and compares them with other methods. Section 4 discusses these results. Finally, Section 5 summarizes the conclusions of this study.

## 2. Materials and Methods

### 2.1. Datasets

In this study, we utilized three public datasets, the EEGdenoiseNet dataset [70], the MIT-BIH Arrhythmia Database [71], and a semi-simulated EEG/EOG dataset [72], to evaluate the performance of the proposed network according to previous research [63,65,67,68,69,73].

The EEGdenoiseNet dataset is a publicly available, structured dataset designed for training and testing deep learning-based denoising models. It also facilitates performance comparisons among different models. This dataset comprises 4514 clean EEG segments, 3400 ocular artifact (EOG) segments, and 5598 muscular artifact (EMG) segments. The clean EEG, pure EOG, and pure EMG segments were sourced from several publicly available data repositories [74,75,76,77,78,79,80,81]. The raw EEG data were collected from 52 participants performing both real and imagined left- and right-hand movement tasks, with 64-channel EEG recorded at a 512 Hz sampling frequency. Clean EEG signals were obtained by resampling to 256 Hz and processed by ICLabel [82]. For EOG segments, multiple open-access EEG datasets with additional EOG channels were used [75,76,77,78,79,81], where horizontal and vertical raw EOG signals were resampled to 256 Hz. For EMG segments, a facial EMG dataset was utilized [80], with raw EMG signals resampled to 512 Hz. All three categories of signals (EEG, EOG, and EMG) were segmented into standardized 2 s, one-dimensional segments according to the previous knowledge. Finally, each segment was visually checked by experts to ensure they are clean and usable.

The MIT-BIH Arrhythmia Database consists of data from 47 subjects monitored by the BIH Arrhythmia Laboratory from 1975 to 1979. It includes 48 half-hour excerpts of two-channel ambulatory ECG recordings with a sampling rate of 360 Hz. Of these, 23 recordings were randomly selected from a set of 4000 24 h ambulatory ECG recordings collected from a mixed population of inpatients (about 60%) and outpatients (about 40%) at Boston’s Beth Israel Hospital. The remaining 25 recordings were deliberately chosen from the same set to capture less common but clinically significant arrhythmias that might not be well represented in a small random sample. These recordings were digitized at 360 samples per second per channel with an 11-bit resolution over a 10 mV range. Two or more cardiologists independently annotated each record, and any disagreements were resolved to create computer-readable reference annotations for each beat, resulting in approximately 110,000 annotations included in the database.

The semi-simulated EEG/EOG dataset includes 54 recordings, each lasting 30 s. The pure EEG signals, contaminated EEG signals, and EOG artifacts are provided in this dataset. EEG data were collected from 27 healthy subjects during an eyes-closed session, with a sampling frequency of 200 Hz. The signals were band-pass filtered at 0.5–40 Hz and notch filtered at 50 Hz. EOG signals were obtained from the same subjects, during an eyes-opened condition, using four electrodes placed above and below the left eye and another two on the outer canthi of each eye. These EOG signals were band-pass filtered at 0.5–5 Hz. The artifact-free EEG signals are manually contaminated with EOG artifacts based on the model proposed by Elbert et al. [83], creating the semi-simulated EEG/EOG dataset.

In our study, pure EMG signals are sourced from the EEGdenoiseNet dataset, pure EOG signals are from both the EEGdenoiseNet and the semi-simulated EEG/EOG dataset, and pure ECG signals are taken from the MIT-BIH Arrhythmia Database. Clean EEG signals are derived from all three datasets. These clean EEG signals serve as the ground truth, while four types of artifacts (EMG, EOG, ECG, and mixed (EOG + EMG)) are linearly combined with the clean EEG signal to produce contaminated EEG recordings.

For consistency, all datasets were resampled at 512 Hz and segmented into one-dimensional segments of 2 s each. Specifically, 3400 pairs of EEG and EOG segments, 5600 pairs of EEG and EMG segments, 3600 pairs of EEG and ECG segments, and 3400 pairs of EEG and mixed artifact (EOG + EMG) segments were used to generate contaminated EEG signals according to Equation (1).

(1) $$y=x+\lambda \cdot n_{t}$$

(2) $$SNR=10\;log\frac{RMS\left(x\right)}{RMS\left(\lambda \cdot n_{t}\right)}$$

(3) $$RMS\left(g\right)=\sqrt{\frac{1}{N}\sum_{i=1}^{N}g_{i}^{2}}$$

where x represents the pure EEG signal as the ground truth (EEG_P_), while y represents the contaminated EEG (EEG_C_), and $n_{t}$ denotes artifacts (e.g., EMG, ECG, EOG, and mixed). The parameter λ is used to regulate the signal-to-noise ratio (SNR) in the contaminated EEG signal, where $\lambda \cdot n_{t}$ denotes the noise components of EEG_C_. RMS refers to the root-mean-squared value. N indicates the number of temporal samples in the segment $g_{i}$, where $g_{i}$ represents the *i*-th sample of a segment g.

For the training set, 80% of the segments were randomly selected from each EEG and artifact pair. Another 10% of the segments from each pair were selected randomly for the test set, while the remaining 10% formed the validation set. Within the training set, pure EEG segments and artifact segments were randomly paired at varying SNR levels ranging from −7 to 2 dB. For both the test and the validation sets, each artifact segment was paired with a pure EEG segment, with mixing conducted once at each of the ten SNR levels (−7, −6, −5, −4, −3, −2, −1, 0, 1, and 2 dB).

### 2.2. Methods

In this study, we introduce DHCT-GAN, a dual-branch hybrid CNN–Transformer network for EEG denoising.

#### 2.2.1. Pseudo Code

See Algorithm 1.
**Algorithm 1** EEG Denoising**Input:** Training datasets (x; y), number of adversarial training iterations T, number of training iterations for the denoising network K, mini-batch size M, and early stopping criterion C. 1: Randomly initialize the generator parameters θ and the parameters of the three discriminators: Φ1 Φ2, Φ2 2:  **for** t ← 1 to T do 3:       for k ← 1 to K do 4:          Sample M instances{x^(m)^, y^(m)^} from the training datasets              //G is the overall generator network, G_1_ generates clean EEG signals, and G_2_ generates noise signals 5:          Compute the loss and gradients for the discriminator network D_1_ 6:          Update the parameters of discriminator network D_1_ 7:          Compute the loss and gradients for the discriminator network D_2_ 8:          Update the parameters of discriminator network D_2_ 9:          Compute the loss and gradients for the discriminator network D_3_ 10:         Update the parameters of discriminator network D_3_ 11:         Compute the loss and gradients for the generator G 12:         Update the parameters of generator network G with θ      **end for** 13:       Compute the loss of generator network G on the validation set 14:       If the validation loss increases consecutively more than C times, then stop training; otherwise, continue training  **end for** **Output:** Generator network G(x; θ)

#### 2.2.2. DHCT-GAN Structure

**Figure 1:**
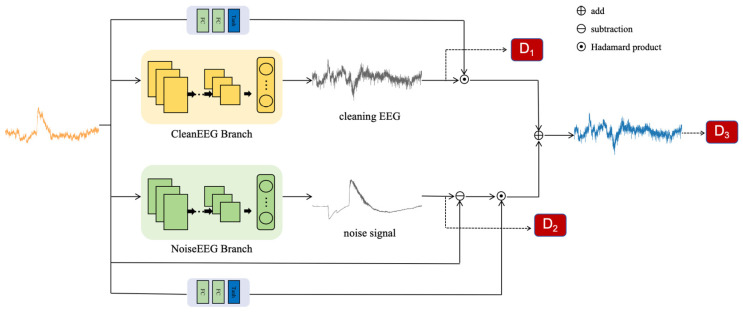
The structure of the proposed network—DHCT-GAN.

In Figure 1, we present DHCT-GAN, a dual-branch network designed for EEG signal denoising. The network architecture comprises a generator (G) and three discriminators (D_1_, D_2_, D_3_). The generator G has two branches and two gating networks: the first branch learns the distribution of clean EEG signals, while the second branch learns the distribution of noise signals. When provided with a sample pair, the generator processes the input X and generates three outputs: an initial prediction of the clean EEG signal, a prediction of the noise signal, and a clean EEG signal derived from the fusion of the two outputs. Subsequently, discriminators D_1_, D_2_, and D_3_ then take these outputs as input and guide the training of the generator through feature loss and adversarial loss.

Gating networks are particularly useful in scenarios involving complex or heterogeneous data. In this study, the gating networks consist of two fully connected layers and use a *tanh* activation function to learn a set of weight coefficients that adaptively fuse the outputs of two branches, generating the constrained clean EEG signal. The reconstructed EEG can be calculated using the following formula:

(4) $$Y_{mask}=f_{gate}(X_{raw})$$

(5) $$Y_{pre}={\;Y_{mask1}⊙Y}_{1}+Y_{mask2}⊙(X_{raw}-Y_{2})$$

where $f_{gate}$ denotes the gating networks, $Y_{pre}$ represents the ultimate predicted clean EEG signal, and $Y_{1}$ and $Y_{2}$ correspond to the outputs of the first and second branches, respectively. $Y_{mask1}$ and $Y_{mask2}$ refer to the mask of the first and second branches, while $X_{raw}$ denotes the original EEG signal.

**Figure 2:**
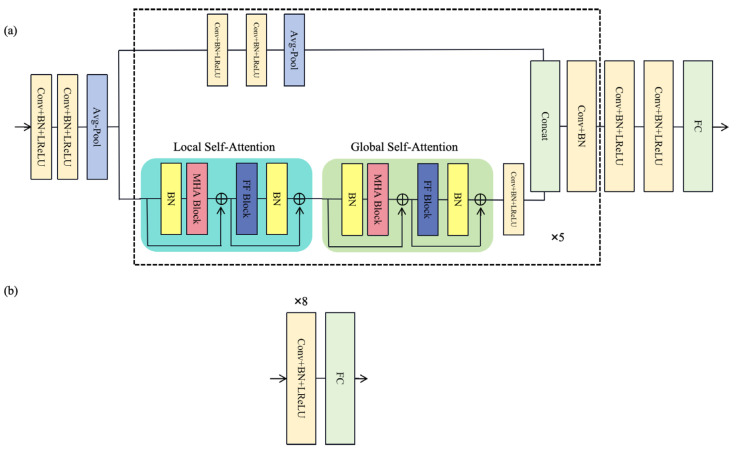
The structure of (**a**) the generator and (**b**) the discriminator.

Each branch generator follows a consistent structure (Figure 2), consisting of three main parts:

1. Preprocessing module: This module converts the one-dimensional signal into high-dimensional (32 dimensions) features by employing two 1-D convolution layers and an average pooling layer to enhance information extraction capabilities.
2. Encoding module: This module consists of 5 CNN-LGTB encoding blocks, which include parallel CNN and Local–Global Transformer Block (LGTB), with information dimensions starting from 64 and doubling incrementally (64, 128, 256, 512, and 1024). The output information of the last encoding block is combined through a feature fusion layer, and after passing through a convolutional layer and batch normalization (BN) layer, it is output as extracted features. The CNN block consists of two convolutional layers with a kernel size of 3, a batch normalization layer, and a Leaky Rectified Linear Unit (LReLU) layer. The LGTB includes a local self-attention module (LSA), a global self-attention module (GSA), and a feedforward module. The local attention module divides the input data into multiple parts (set to 8 blocks in this study), calculates local attention for each part, and then concatenates them to form local attention features. Local self-attention focuses on a limited context, which enables the model to effectively capture local dependencies within the input sequence. In contrast, global self-attention considers the entire sequence, allowing the model to capture long-range dependencies and overall contextual relationships. By integrating both local and global self-attention mechanisms, the model effectively captures a wide range of dependencies, enhancing its ability to understand complex patterns within the data. This combination facilitates a more comprehensive representation of the input, improving performance across various tasks. The output is then passed through a feedforward network to the global attention module, where global attention features are computed. Subsequently, the output undergoes further processing through a feedforward network, a convolutional layer, a batch normalization layer, and an LReLU layer.
3. Decoding module: This module reverts the intermediate features back to the original signal dimension by utilizing two convolutional layers and one fully connected layer to produce denoised signals.

The three discriminators, denoted as D_1_, D_2_, and D_3_, have the same structure as shown in Figure 2a. The input signals undergo processing through M convolutional layers, each layer followed by a batch normalization layer and an activation function layer. In this research, M is defined as 8. The output channels of the convolutional layers are 64, 64, 128, 128, 256, 256, 512, and 512, respectively. The kernel size of each one-dimensional convolutional layer is 3, with a stride of 2 and padding of 1. D_1_ discriminates between real clean signals and clean signals predicted by the first branch, while D_2_ distinguishes between real noisy signals and the noisy signals predicted by the second branch. D_3_ focused on differentiating real clean signals from the clean signals ultimately predicted by the generator. Together, these three discriminators offer additional loss functions to the generator, enhancing the quality of the generated signals.

#### 2.2.3. Loss Function

The loss function employed in this study comprises the mean squared error (MSE) loss for the output of the generator, in addition to the feature loss and adversarial loss for the output of the discriminator.

There are three losses (Loss1, Loss2, Loss3) in the generator; each loss is composed of MSE loss ($L_{mse}$), feature loss ($L_{feat}$), and adversarial loss ($L_{adv}$), with added constraint coefficients. Here, $\lambda_{1}\;$ and $\lambda_{2}$ are the weights of feature loss and adversarial loss.

(6) $$Loss1={L1}_{mse}+\lambda_{1}{L1}_{feat}+\lambda_{2}{L1}_{adv}$$

(7) $$Loss2={L2}_{mse}+\lambda_{1}{L2}_{feat}+\lambda_{2}{L2}_{adv}$$

(8) $$Loss3={L3}_{mse}+\lambda_{1}{L3}_{feat}+\lambda_{2}{L3}_{adv}$$

Total loss is described in Equation (9), where $L_{total}$ is the total loss of the generator, Loss1 and Loss2 are the total loss of the first and second branch, respectively, and Loss3 denotes the loss associated with the final output of the generator.

(9) $$L_{total}=Loss1+Loss2+Loss3$$

MSE loss, the feature loss, and the adversarial loss are expressed as follows:

(10) $$L_{mse}=\frac{1}{N}\sum_{i=1}^{N}{\left(Y_{i}-G\left(X_{i}\right)\right)}^{2}$$

(11) $$L_{feat}=\frac{1}{N}\sum_{i=1}^{N}{\left(\varphi \left(Y_{i}\right)-\varphi \left(G(X_{i})\right)\right)}^{2}$$

(12) $$L_{adv}=\frac{1}{N}\sum_{i=1}^{N}{\left(D\left(G\left(X_{i}\right)\right)-1\right)}^{2}$$

where Y represents the ground truth used during model training and $G\left(X\right)$ denotes the model’s output, which includes the predicted clean signal as well as the noise signal, subscript i represents the *i*-th sample, and φ  denotes the feature from the intermediate convolution layer of the discriminator.

In Equation (10), given the input data X, its denoised prediction $G\left(X\right)$, and the corresponding ground truth clean data Y, the mean squared error (MSE) between $G\left(X\right)$ and Y is computed for each sample. The average of these MSE values across all samples results in a scalar value that quantifies the discrepancy between the predicted and ground truth data.

In Equation (11), the feature representations of both the predicted values $G\left(X\right)$ and the ground truth Y are extracted using the discriminator. The feature loss is calculated by computing the MSE between the intermediate features of $G\left(X\right)$, ($\varphi G\left(X\right)$) and Y ($\varphi G\left(Y\right)$) derived from the discriminator. This yields a scalar value that quantifies the difference between the predictions and the ground truth in the feature space.

In Equation (12), the predicted values $G\left(X\right)$ are passed through the discriminator to obtain the output probability, which represents the likelihood of the input being classified as real. To quantify the gap between the predicted values and real samples in the discriminator, the squared difference between the output probability and 1 is computed. This produces a scalar value that reflects the confidence difference in the discriminator’s classification of the predictions.

The discriminators were trained using the discriminator loss ($L_{D}$) to enhance the accuracy in distinguishing $G\left(X\right)$ and Y.

(13) $$L_{D}=\frac{1}{N}\sum_{i=1}^{N}\left(\frac{1}{2}({D\left(G\left(X\right)\right))}^{2}+\frac{1}{2}({D\left(Y\right)-1)}^{2}\right)$$

#### 2.2.4. Model Training and Implementation

During the training process, generators (G) and discriminators (D_1_, D_2_, D_3_) are trained alternately. Initially, the parameters of the generator G are kept constant while the discriminators (D_1_, D_2_, and D_3_) are trained individually. Each discriminator receives different inputs: D_1_ is provided with the clean EEG signal generated by the first branch of the generator, D_2_ receives the noise signal generated by the second branch of the generator, and D_3_ receives the denoised EEG signal generated by the generator. Subsequently, the parameters of each discriminator are independently updated based on their respective loss function. Following this, the discriminator parameters are fixed, and the original signal (X) is input into the generator G, with the parameters of G being updated according to the total loss of the generator. Early stopping is implemented during the training phase to prevent model overfitting by monitoring the model’s performance metric (MSE updated with each epoch) on the validation dataset. If no significant improvement is observed over a certain number of epochs, the training is stopped. The model with the best performance is then selected for deployment. Notably, the incorporation of three discriminators enhances the stability of the model by not only computing the loss for the overall output but also evaluating the loss for each branch separately. During the testing phase, the original signal (X) is input into the generator G, and the denoised EEG signal predicted by the generator is used as the final output.

The training process of GANs is often unstable and prone to mode collapse. To mitigate this, we adjust the learning rate to 0.001. The Adam optimizer is employed for model optimization, with the generator’s Adam parameters set to β1 = 0.5 and β2 = 0.9, and the discriminator’s Adam parameters set to β1 = 0.9 and β2 = 0.999. The learning rate is fixed at 0.0001, and the batch size is set to 40. The maximum number of training epochs is limited to 1000. All experiments are conducted using Python 3.7.13 and the PyTorch 2.1.0 framework on an NVIDIA RTX 4090 GPU, manufactured by NVIDIA Corporation in Santa Clara, CA, USA.

#### 2.2.5. Evaluation Metrics

Several objective measures are used to complete the quantitative performance analysis, including Relative Root Mean Square Error (*RRMSE*) in the temporal domain (*RRMSE_t_*), *RRMSE* in the spectral domain (*RRMSE_f_*), the average correlation coefficient (*CC*), the temporal percentage reduction in artifacts from the EEG_C_ (η), the structural similarity index measure (*SSIM*), and mutual information (*MI*). *RRMSE_t_* and *RRMSE_f_* define the energy error of the signal in the temporal and spectral domains, respectively. Smaller values indicate better artifact removal performance. *CC* measures the similarity between the predicted EEG and the ground truth EEG; a higher *CC* value indicates greater similarity, while a lower value indicates less agreement. To assess the statistical significance of the *CC*, a p-value is calculated. Typically, a small p-value (<0.05) suggests that the observed correlation is statistically significant. A higher η value indicates that more artifacts have been removed from the contaminated EEG signal, resulting in a cleaner and more accurate EEG signal. *SSIM* is a measure of signal similarity; a higher *SSIM* value indicates that the predicted EEG is more similar to the ground truth EEG. *MI* measures the dependency between two signals, with larger *MI* values signifying stronger mutual dependence or correlation between the predicted EEG and the ground truth. *RRMSE_t_*, *RRMSE_f_*, *CC*, η, *SSIM* and *MI* are defined as follows:

(14) $${RRMSE}_{t}=\frac{RMS\left(f\left(y\right)-x\right)}{RMS\left(x\right)}$$

(15) $${RRMSE}_{f}=\frac{RMS\left(PSD\left(f\left(y\right)\right)-PSD\left(x\right)\right)}{RMS\left(PSD\left(x\right)\right)}\;$$

(16) $$CC=\frac{Cov\left(f\left(y\right),x\right)}{\sqrt{Var\left(f\left(y\right)\right)Var\left(x\right)}}$$

(17) $$\eta =\left(\frac{1-CC}{1-{CC}_{b}}\right)*100$$

(18) $${CC}_{b}=\frac{Cov\left(y,x\right)}{\sqrt{Var\left(y\right)Var\left(x\right)}}\;$$

(19) $$SSIM=\frac{\left(2\mu_{x}\mu_{f\left(y\right)}+C_{1}\right)\left(2Cov\left(f\left(y\right),x\right)+C_{2}\right)}{\left(\mu_{x}^{2}+\mu_{f\left(y\right)}^{2}+C_{1}\right)\left(Var\left(x\right)+Var\left(f\left(y\right)\right)+C_{2}\right)}$$

(20) $$MI=\int_{-\infty }^{\infty }p(x,f\left(y\right))\log\left(\frac{p\left(x,f\left(y\right)\right)}{p\left(x\right)p\left(f\left(y\right)\right)}\right)dx\;df(y)$$

Here, the denoised EEG signals are represented by $f\left(y\right)$, the PSD is the power spectral density computed using the Periodogram method, and the functions Cov and Var denote the covariance and variance of the signal, respectively. ${CC}_{b}$ is used to represent the time-domain correlation coefficient between EEG_C_ and EEG_P_ segments. $\mu_{x}$ and $\mu_{f\left(y\right)}$ are the average values of the pure EEG signals (x) and the denoised EEG signals ($f\left(y\right)$). $C_{1}$ and $C_{2}$ are small constants set to avoid the denominator being zero; usually, $C_{1}=10^{-4}$ and $C_{2}=10^{-4}$. $p(x,f\left(y\right))$ is the joint probability density function of x and $f\left(y\right)$, and $p\left(x\right)$ and $p\left(f(y)\right)$ are the marginal probability density function of x and $f\left(y\right)$, respectively.

## 3. Results

### 3.1. Performance Evaluation

In order to assess the efficacy of DHCT-GAN, we conducted a comparative analysis with five state-of-the-art deep learning-based EEG denoising techniques: simple convolutional neural network (SimpleCNN), long short-term memory network (LSTM) [84], dual-scale CNN-LSTM model (DuoCL) [53], a GAN-guided parallel CNN and Transformer network (GCTNet) [68], and EEGDiR [57]. The comparison experiments were carried out using the same training and testing settings, for example, the same data separation and same batch size.

**Figure 3:**
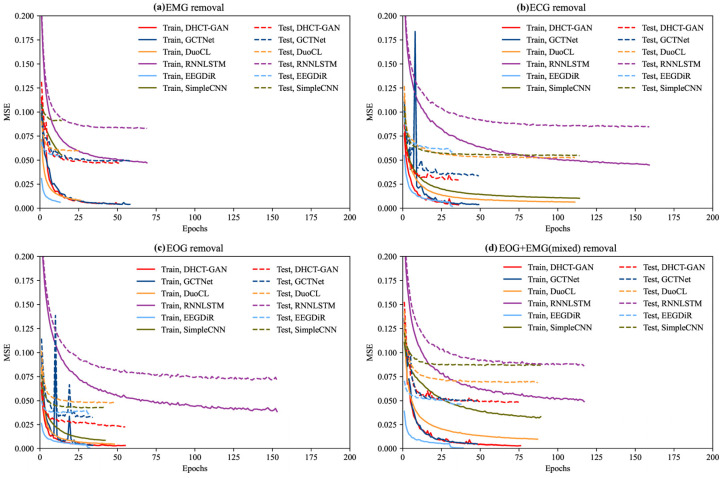
The MSE loss curve of DHCT-GAN, GCTNet, DuoCL, EEGDiR, RNNLSTM, and SimpleCNN in the training (solid lines) and testing (dashed lines) process in (**a**) EMG removal, (**b**) ECG removal, (**c**) EOG removal, and (**d**) EOG + EMG (mixed) removal tasks.

Figure 3 illustrates the variation in MSE as a function of the number of iterations (epochs) during the training and testing process. Overall, the training and testing MSEs for each method generally decrease with increasing iterations, eventually reaching a stable state. In the task of EMG artifact removal, DHCT-GAN, DuoCL, GCTNet, and EEGDiR demonstrate similar performance on the training set, achieving the lowest MSE values. Among these, EGDiR exhibits the fastest convergence, while DHCT-GAN achieves the lowest MSE on the testing set. Notably, DHCT-GAN shows superior generalization ability among these four models, with the smallest MSE difference between the training and testing sets. This highlights the robustness and efficiency of DHCT-GAN for EMG artifact removal tasks. In the task of ECG artifact removal, the MSE values of DHCT-GAN, DuoCL, GCTNet, and EEGDiR are similar in the training set. Among these, EEGDiR achieves the lowest MSE on the training set, and exhibits the fastest convergence (converges around 25 epochs). However, DHCT-GAN achieves the lowest MSE on the testing set, demonstrating a good generalization. In the task of EOG artifact removal, DHCT-GAN, EEGDiR, and GCTNet demonstrate the best performance on the training set, achieving the lowest MSE values. DHCT-GAN achieves the lowest MSE on the testing set, outperforming the other methods in terms of accuracy and generalization. Among these, GCTNet and EEGDiR exhibit the fastest convergence, but GCTNet’s convergence curve exhibits greater fluctuations, indicating reduced stability. In the tasks of EMG, ECG, and EOG artifact removal, EEGDiR fits the training set well but performs poorly on the testing set. In the task of EOG and EMG mixed artifact removal, EEGDiR demonstrates the best performance on the training and testing sets. DHCT-GAN converges at approximately 75 epochs, while GCTNet converges at around 50 epochs. DHCT-GAN and GCTNet achieve comparable performance.

**Figure 4:**
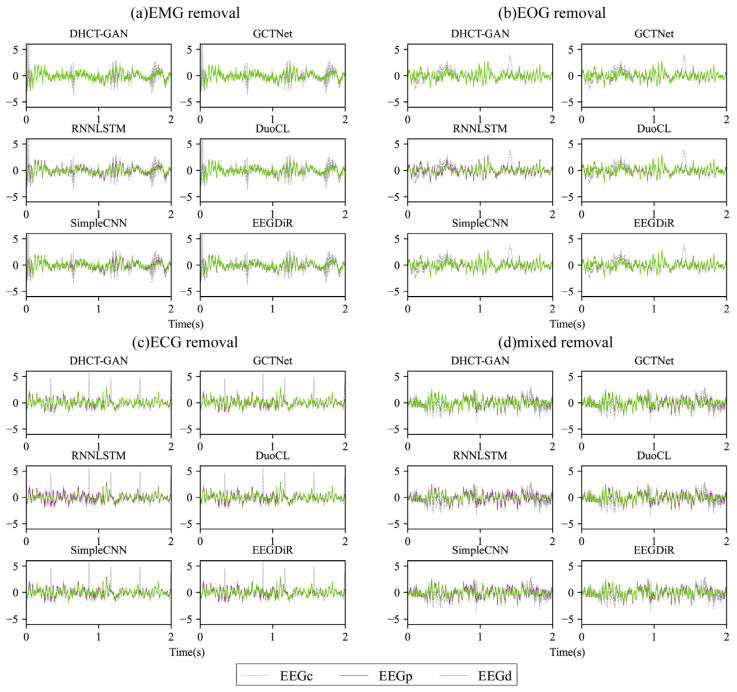
The exemplary waveform obtained after removing (**a**) EMG, (**b**) ECG, (**c**) EOG, and (**d**) EOG + EMG (mixed) artifacts using various networks from contaminated EEG signals with an SNR of 0 dB. EEG_P_ represents the pure EEG signal considered the ground truth, EEG_C_ represents the contaminated EEG signal, and EEG_D_ represents the denoised EEG signal.

The exemplary waveforms obtained after removing four types of artifacts are displayed in Figure 4. In comparison to EEG_C_, the waveform of EEG_D_ from various networks shows significant changes (Figure 4). It is apparent that DHCT-GAN, GCTNet, DuoCL, and EEGDiR demonstrate notable effectiveness in mitigating EMG and EOG artifacts in the contaminated signals. While the EEG_D_ waveform exhibits slight variations from the EEG_P_ waveform, these two waveforms are highly similar and show considerable overlap, indicating successful EEG signal reconstruction. In ECG and mixed artifact removal tasks, DHCT-GAN and GCTNet demonstrate better performance than DuoCL and EEGDiR. On the other hand, RNNLSTM and SimpleCNN display significant signal distortion in the reconstructed data when compared to the original contaminated EEG signals after artifact removal. Moreover, the reconstructed waveforms from GCTNet, DuoCL, and EEGDiR exhibit instances of peak amplitude overflow and local waveform offset, indicating a loss of specific waveform details. These findings illustrate that DHCT-GAN can efficiently eliminate ECG, EOG, EMG, and mixed (EOG + EMG) artifacts and reconstruct EEG signals.

**Figure 5:**
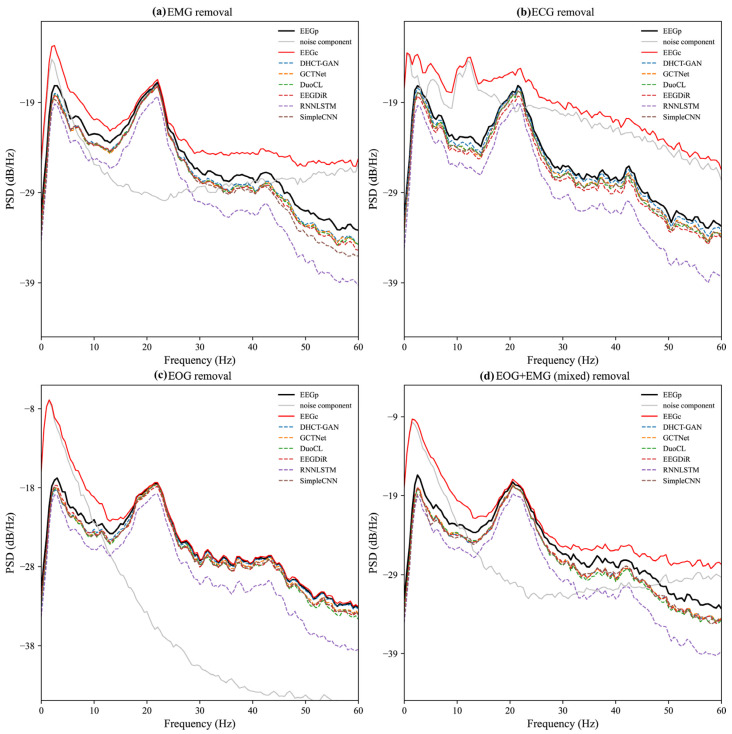
PSD results of the pure EEG signal (EEG_P_, black solid line), the contaminated EEG signal (EEG_C_, red solid line), and the noise component (EEG_C_-EEG_P_, gray solid line). PSD results obtained after removing (**a**) EMG, (**b**) ECG, (**c**) EOG, and (**d**) EOG + EMG (mixed) artifacts from the contaminated EEG data by various networks (dashed lines).

The PSD results of the noise component (EEG_C_-EEG_P_) are displayed in Figure 5. In the EMG artifact removal task, the PSD of the noise component shows substantial values across all frequencies, indicating that the energy distribution of EMG artifacts is relatively uniform. In the mixed artifact removal task, the PSD pattern of the noise component closely resembles that of EMG artifacts, suggesting that the characteristics of mixed artifacts (EOG + EMG) are primarily dominated by EMG. The PSD of EOG and ECG artifacts is primarily concentrated in low-frequency (0–20 Hz) ranges and decays rapidly thereafter (Figure 5b,c).

The PSD results after artifact removal, the pure EEG signal (EEG_P_), and the contaminated EEG signal (EEG_C_) are also presented in Figure 5. A greater divergence between the PSD line and the red line (EEG_C_) indicates that more artifacts in the frequency domain have been removed. Conversely, a closer alignment of the PSD line with the black line (EEG_P_) indicates minimal loss in the frequency domain after artifact removal. In the task of EMG and mixed artifact removal, the PSD lines of DHCT-GAN, DuoCL, EEGDiR, GCTNet, and SimpleCNN align closely with the black line across certain frequencies (e.g., 0–5 Hz, 20–25 Hz), demonstrating their strong EMG denoising performance. In the task of ECG and EOG artifact removal, the PSD lines of DHCT-GAN, GCTNet, DuoCL, EEGDiR, and SimpleCNN also exhibit close alignment with EEG_P_ across all frequencies, indicating effective denoising capabilities. In contrast, the PSD lines of RNNLSTM exhibit significant overlap with EEG_C_ across all artifact types, revealing substantial frequency domain information loss and highlighting their comparatively poor performance.

In order to more accurately measure the effectiveness of these models in removing artifacts, we provide quantitative results for six performance metrics (*RRMSE_f_*, *RRMSE_t_*, *CC*, η, *SSIM* and *MI*) by averaging the values across all SNR levels in Table 1. Lower values of *RRMSE_t_* and *RRMSE_f_* indicate that the denoised EEG signals maintain minimal deviation in both the time and frequency domains. Likewise, higher values of *CC*, η, *SSIM* and *MI* indicate that more authentic EEG information is retained while effectively eliminating artifacts. To assess the statistical significance of the *CC*, a p-value is calculated. Typically, A small p-value (<0.05) suggests that the observed correlation is statistically significant.

Compared to the other five models, DHCT-GAN achieves superior results across all metrics. In the EMG artifact removal task, DHCT-GAN demonstrates the minimum values for *RRMSE_t_* (0.3918) and *RRMSE_f_* (0.2837), as well as the maximum values for *CC* (0.9201), *η* (82.35%), *SSIM* (0.7167), and *MI* (1.0031) among six models. In the EOG artifact removal task, DHCT-GAN exhibits the lowest *RRMSE_t_* (0.2734) and *RRMSE_f_* (0.2024), and the highest *CC* (0.9624), *η* (91.80%), *SSIM* (0.8402), and *MI* (1.4290). When addressing ECG artifacts, DHCT-GAN outperforms all other models with the lowest *RRMSE_t_* (0.3111) and *RRMSE_f_* (0.2285), and the highest *CC* (0.9504), *η* (88.97%), *SSIM* (0.7796), and *MI* (1.2309). Moreover, DHCT-GAN surpasses the comparison method in removing mixed artifacts, displaying the lowest *RRMSE_t_* (0.3975) and *RRMSE_f_* (0.2904), and the highest CC (0.9184), *η* (82.07%), *SSIM* (0.6996) and *MI* (1.0159).

Among the four types of artifacts, our network demonstrates superior performance in removing EOG artifacts, with the ability to eliminate ECG artifacts ranking second. The effectiveness of our network in eliminating EMG and mixed artifacts is comparable. Notably, judging from the metrics of *RRMSE_f_*, *RRMSE_t_*, *CC*, *η*, *SSIM,* and *MI*, GCTNet is closely aligned with our work in tasks related to removing ECG and mixed artifacts, while EEGDiR is closely aligned with our work in removing mixed artifacts. At the same time, the p-values corresponding to the correlation coefficients are less than 0.05, which indicates that the relationship between prediction signals and the true signals is statistically significant.

**Figure 6:**
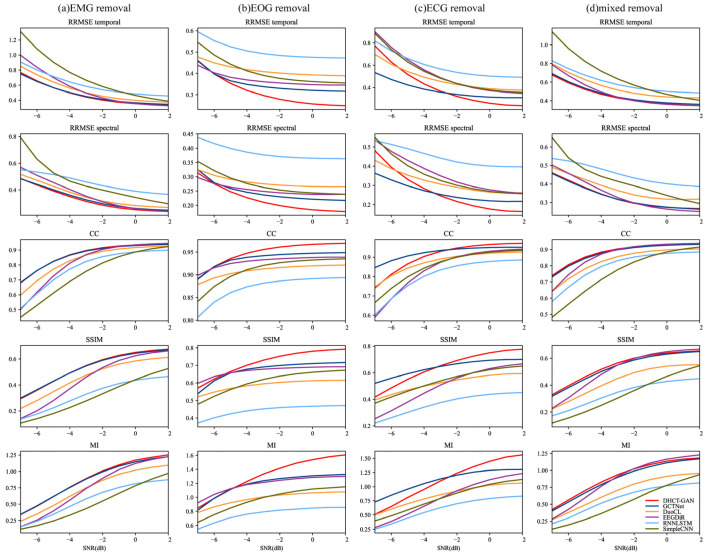
Performance metric estimates (*RRMSE_f_*, *RRMSE_t_*, *CC*, *SSIM*, *MI*) at various SNR levels (from −7 to 2 dB) in (**a**) EMG (**b**) ECG, (**c**) EOG and (**d**) EOG + EMG (mixed) artifact removal tasks.

To comprehensively compare these networks, performance metrics are evaluated at various SNR levels, and the Kruskal–Wallis H test is conducted. As illustrated in Figure 6, the performance of artifact removal generally improves with increasing SNR levels. Our network achieves the highest *CC*, *SSIM*, and *MI* values, and the lowest *RRMSE_f_* and *RRMSE_t_* values for EOG artifact removal tasks at SNR levels from −6 to 2 dB (*p* < 0.05 for all metrics). In contrast, for EMG and mixed artifacts, both our network and GCTNet demonstrate similar performance across all SNR levels, with our network showing a slight advantage (*p* < 0.05 for all metrics), consistent with the results in Table 1. In the case of ECG artifact removal, GCTNet outperforms our network at SNR levels ranging from −7 to −3 dB (*p* < 0.05 for all metrics). However, our network exhibits superior performance in removing ECG artifacts at SNR levels from −2 to 2 dB (*p* < 0.05 for all metrics).

The results obtained by DHCT-GAN for different SNR levels of ECG artifacts show greater variability, while those for EOG, EMG, and mixed artifacts show less variability. These findings suggest that the extent of ECG artifact contamination has a more pronounced impact on the network’s performance compared to the other three types of artifacts.

### 3.2. Ablation Study

In DHCT-GAN, the first branch, referred to as the clean-learning branch, directly learns the distribution of clean EEG signals, while the second branch, referred to as the noise-learning branch, learns the characteristics of noise signals. In order to explore the necessity of using a dual-branch structure, ablation studies were conducted as follows: (1) using only the clean-learning branch (clean), (2) using only the noise-learning branch (noise), and (3) using the proposed network (combining the noise-learning branch and the clean-learning branch, DHCT-GAN).

In this study, Transformers are integral to the DHCT-GAN architecture, but their role in the hybrid EEG artifact removal process is not sufficiently explored. In order to clarify the contribution of the Transformer component, we designed ablation experiments as follows: (1) remove the Transformer module from the proposed network (No-Transformer), (2) replace the Transformer module with a simple self-attention mechanism module (self-attention), and (3) the proposed network (hybrid CNN–Transformer modules, DHCT-GAN).

The results of the ablation experiments are presented in Table 2.

The DHCT-GAN integrates a noise learning branch and a clean learning branch. Compared to using the clean learning branch alone or the noise learning branch alone, the proposed network demonstrates stronger artifact removal performance on different types of artifacts. Additionally, the clean learning branch performs well in removing ECG, EMG, and mixed artifacts, while the noise learning branch is more effective in removing EOG artifacts compared to the clean learning branch and other networks. Furthermore, when dealing with EMG and mixed artifacts, using the noise learning branch alone leads to significantly poorer denoising performance. This may be due to the fact that the noise learning branch is more prone to learning the distribution characteristics of low-frequency noise.

For four types of artifact removal tasks, the network without the Transformer module (No-Transformer) performs better than the network using a hybrid CNN and self-attention mechanism module. Compared to the network without the Transformer module or using the self-attention mechanism module, the proposed network demonstrates stronger denoising performance. These results emphasize the contribution of the Transformer component in our network.

**Figure 7:**
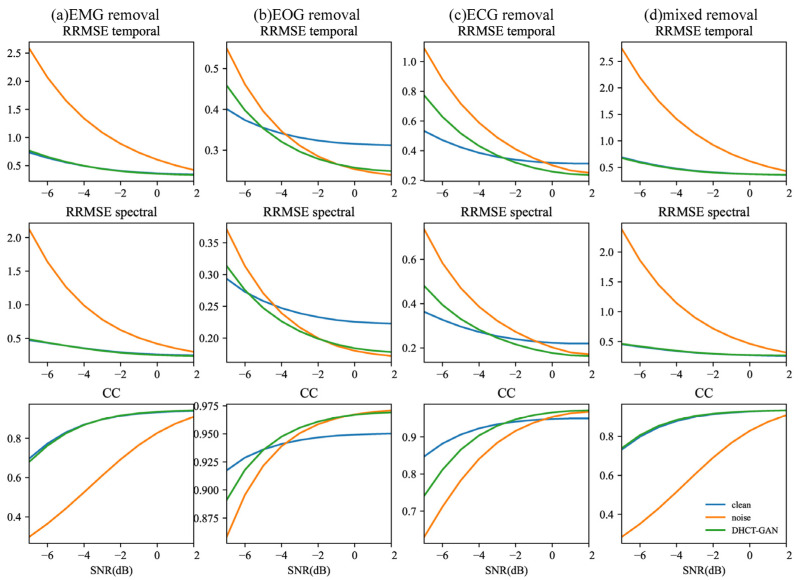
The results of the ablation experiment (using clean branch only or noise branch only) conducted at varying SNR levels (from −7 to 2 dB) in (**a**) EMG, (**b**) ECG, (**c**) EOG, and (**d**) EOG + EMG (mixed) artifact removal tasks.

**Figure 8:**
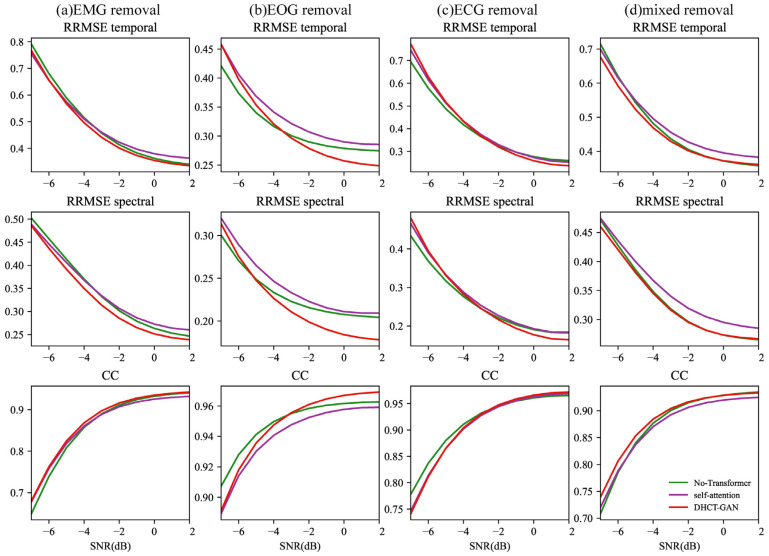
The results of the ablation experiment (remove the Transformer module or replace the Transformer module with a self-attention mechanism module) conducted at varying SNR levels (from −7 to 2 dB) in (**a**) EMG, (**b**) ECG, (**c**) EOG, and (**d**) EOG + EMG (mixed) artifact removal tasks.

The study also investigated the performance metrics (RRMSEf, RRMSEt, CC) of the ablation experiment at various SNR levels, as shown in Figure 7 and Figure 8. The Kruskal–Wallis H test was conducted, and p < 0.05 for all metrics. In Figure 7, for tasks involving EMG and mixed artifact removal, the performance of the DHCT-GAN and the clean-learning branch alone exhibits similar performance at equivalent SNR levels, and both approaches outperform using the noise learning branch alone across all SNR levels. In the case of EOG removal, the clean learning branch alone demonstrates superior performance between −7 and −5 dB SNR levels, while the DHCT-GAN achieves the best performance between −5 and −1 dB SNR levels. However, the performance of the DHCT-GAN is slightly inferior to using the noise learning branch alone from 0 to 2 dB SNR levels. Regarding ECG removal, the clean learning branch alone performs best between −7 and −4 dB SNR levels. In comparison to utilizing the clean learning branch alone, the noise learning branch alone demonstrates enhanced ECG removal performance within the 0 to 2 dB SNR range, and the DHCT-GAN achieves optimal performance between −2 and 2 dB SNR levels.

In Figure 8, for tasks involving EMG, ECG, and mixed artifact removal, the network without the Transformer module (CNN module only) and the network using the hybrid CNN and self-attention mechanism module exhibit similar performance at equivalent SNR levels. The performance of DHCT-GAN is slightly better than the network without the Transformer module (CNN module only) as well as the network using the hybrid CNN and self-attention mechanism module at all SNR levels in EMG and mixed artifact removal tasks. In the case of EOG removal, DHCT-GAN performs best between −3 and 2 dB SNR levels, and the network without the Transformer module (CNN module only) performs better than the network using hybrid CNN and self-attention mechanism module between −7 and −5 dB SNR levels. In the ECG artifact removal task, the network without the Transformer module performs best between −7 and −4 dB SNR levels.

## 4. Discussion

In the analysis and applications of EEG data, removing artifacts is crucial. In this study, we propose DHCT-GAN, a dual-branch network that integrates cross-domain knowledge for EEG denoising. To improve the EEG denoising performance, we made some key improvements to the model architecture. Firstly, we developed the dual-branch architecture and gated network module to incorporate cross-domain knowledge of both EEG signals and artifacts, overcoming the limitations of traditional clean-signal-only methods. Secondly, the network leverages the complementary strengths of CNNs and Transformers to capture both spatial and temporal characteristics of EEG signals and artifacts, enhancing the model’s adaptability to complex signal dynamics. Thirdly, by integrating both global and local Transformers, the model can capture long-term and short-term dependencies in the data, improving feature robustness and interpretability. Furthermore, the use of multiple discriminators in the GAN framework improves model stability.

Through waveform analysis, power spectral density examination, and quantitative evaluation of six performance metrics, DHCT-GAN demonstrates its ability to preserve intrinsic brain activity after artifact removal. Compared with five state-of-the-art deep learning-based EEG denoising works (SimpleCNN, LSTM, DuoCL, GCTNet, and EEGDiR), DHCT-GAN also demonstrates superior performance in preserving the physiological features of EEG while effectively mitigating multiple types of artifacts. Ablation experiments further prove that the overall denoising performance of DHCT-GAN surpasses methods focusing solely on learning clean EEG signals. In general, DHCT-GAN preserves essential neurophysiological characteristics while significantly reducing interference from different artifacts. This improvement not only enhances the diagnostic accuracy of EEG in clinical environments but also increases the robustness and reliability of BCI systems in decoding user intent.

However, this study still has some limitations. First, DHCT-GAN has shown promising performance on several public datasets, but its performance on real data was not explored in this study, facing challenges with generalization. Second, in this study, all EEG and artifacts were segmented into one-dimensional segments, without considering correction amongst channels. Furthermore, due to the dual-branch architecture and the inclusion of a GAN structure, the DHCT-GAN model has relatively slow inference speeds, requiring more computational power and memory than other simple networks.

Therefore, future works will focus on enhancing the generalization capabilities of DHCT-GAN. Our study primarily focuses on the time domain, and future work could consider incorporating frequency domain information to enhance the performance of DHCT-GAN. Future work could also explore how to integrate the denoising model with existing EEG devices and systems, which could be achieved by developing APIs or utilizing standard interfaces such as OpenEEG or BIDS to facilitate data transmission and interaction between the devices and the model. Furthermore, given the potential of graph neural networks for denoising tasks, future research could also explore their application in this context.

## 5. Conclusions

This study proposes DHCT-GAN, a dual-branch network that integrates cross-domain knowledge for enhancing the denoising of EEG signals. Through waveform analysis, power spectral density examination, and quantitative evaluation using performance metrics, DHCT-GAN demonstrates its superior ability to preserve intrinsic brain activity after removing various types of artifacts compared to recent state-of-the-art methods. Furthermore, ablation experiments show that the hybrid model surpasses single-branch models in artifact removal, underscoring the crucial role of artifact knowledge constraints in improving denoising effectiveness. In conclusion, this study establishes DHCT-GAN as a robust method for artifact removal in RRG signals, effectively leveraging cross-domain knowledge to achieve state-of-the-art results.

## Figures and Tables

**Table 1:** Average performance of artifact removal for EMG, EOG, ECG, and mixed (EOG + EMG) signals by all networks.

Artifact Types	Metrics	DuoCL	GCTNet	EEGDiR	LSTM	SimpleCNN	DHCT-GAN
EMG	RRMSE_t_	0.4407	0.4003	0.4218	0.5213	0.5457	0.3918
	RRMSE_f_	0.3168	0.2942	0.3131	0.4270	0.3934	0.2837
	CC *	0.8977	0.9166	0.9069	0.8628	0.8390	0.9201
	η	77.40%	81.58%	79.43%	69.71%	64.44%	82.35%
	SSIM	0.6600	0.7137	0.6528	0.5627	0.5091	0.7167
	MI	0.8590	0.9898	0.8700	0.6792	0.5880	1.0031
EOG	RRMSEt	0.3985	0.3285	0.3533	0.4832	0.3743	0.2734
	RRMSEf	0.2755	0.2341	0.2473	0.3804	0.2612	0.2024
	CC *	0.9173	0.9448	0.9359	0.8872	0.9275	0.9624
	η	81.95%	87.96%	86.02%	75.40%	84.18%	91.80%
	SSIM	0.7524	0.8109	0.8027	0.6627	0.7444	0.8402
	MI	1.0673	1.2804	1.2787	0.8375	1.0688	1.4290
ECG	RRMSEt	0.4158	0.3322	0.4228	0.5281	0.4246	0.3111
	RRMSEf	0.2936	0.2446	0.3185	0.4198	0.3050	0.2285
	CC *	0.9095	0.9437	0.9076	0.8606	0.9054	0.9504
	η	79.87%	87.49%	79.46%	70.00%	78.97%	88.97%
	SSIM	0.6960	0.7840	0.6771	0.5739	0.6767	0.7796
	MI	0.9506	1.2157	0.9239	0.7038	0.9043	1.2309
EOG + EMG (mixed)	RRMSEt	0.4724	0.4025	0.3978	0.5280	0.5301	0.3975
	RRMSEf	0.3334	0.2946	0.2951	0.4251	0.3891	0.2904
	CC *	0.8814	0.9160	0.9184	0.8588	0.8480	0.9184
	η	73.94%	81.53%	82.06%	68.96%	66.59%	82.07%
	SSIM	0.6046	0.6899	0.6794	0.5334	0.4899	0.6996
	MI	0.7960	0.9903	0.9891	0.6810	0.6142	1.0159

* All *CC* values are accompanied by *p*-values lower than 0.05.

**Table 2:** Performance metrics for ablation experiments.

Artifact Types	Metrics	Clean	Noise	No-Transformer	Self-Attention	DHCT-GAN
EMG	RRMSEt	0.3958	0.8405	0.4022	0.4134	0.3918
	RRMSEf	0.2904	0.6667	0.2986	0.3046	0.2837
	CC *	0.9186	0.7225	0.9159	0.9109	0.9201
	η	82.02%	38.71%	81.42%	80.32%	82.35%
EOG	RRMSEt	0.3215	0.2785	0.2885	0.3033	0.2734
	RRMSEf	0.2318	0.2009	0.2151	0.2232	0.2024
	CC *	0.9474	0.9605	0.9587	0.9536	0.9624
	η	88.51%	91.37%	90.99%	89.88%	91.80%
ECG	RRMSEt	0.3356	0.3907	0.3169	0.3199	0.3111
	RRMSEf	0.2452	0.2883	0.2307	0.2370	0.2285
	CC *	0.9423	0.9243	0.9486	0.9477	0.9504
	η	87.18%	83.17%	88.57%	88.37%	88.97%
EOG + EMG (mixed)	RRMSEt	0.4000	0.8771	0.4003	0.4206	0.3975
	RRMSEf	0.2908	0.7647	0.2943	0.3115	0.2904
	CC *	0.9167	0.7159	0.9171	0.9086	0.9184
	η	81.70%	37.61%	81.79%	79.93%	82.07%

* All *CC* values are accompanied by *p*-values lower than 0.05.

## Data Availability

The original data presented in the study are openly available. EEGdenoiseNet dataset: https://github.com/ncclabsustech/EEGdenoiseNet, accessed on 11 December 2023; MIT-BIH Arrhythmia Database: https://physionet.org/content/mitdb/1.0.0/, accessed on 1 January 2024; the semi-simulated EEG/EOG dataset: https://data.mendeley.com/datasets/wb6yvr725d/1, accessed on 20 January 2024.

## Glossary

**Transformer:** A deep learning architecture widely used in natural language processing and computer vision tasks. It relies on a mechanism called self-attention to process sequential data efficiently and capture long-range dependencies. Transformers have revolutionized fields such as machine translation and image recognition. **GAN (Generative Adversarial Network):** A type of machine learning model composed of two neural networks, a generator and a discriminator, that compete against each other. The generator aims to create data that mimic real samples, while the discriminator evaluates the authenticity of the generated data. This adversarial process improves the generator’s ability to produce realistic outputs. **Adversarial Training:** A training strategy where a model is exposed to adversarial examples—inputs designed to challenge the model’s predictions. This approach improves robustness by teaching the model to handle such challenging scenarios, often used in conjunction with GANs or to defend against attacks in security-sensitive applications.
